# Predicting Virulence of *Fusarium oxysporum* f. sp. *Cubense* Based on the Production of Mycotoxin Using a Linear Regression Model

**DOI:** 10.3390/toxins12040254

**Published:** 2020-04-14

**Authors:** Chuange Shao, Dandan Xiang, Hong Wei, Siwen Liu, Ganjun Yi, Shuxia Lyu, Li Guo, Chunyu Li

**Affiliations:** 1 College of Bioscience and Biotechnology, Shenyang Agricultural University, Shenyang 110866, China; shaochuange@hotmail.com; 2 Institute of Fruit Tree Research, Guangdong Academy of Agricultural Sciences, Key Laboratory of South Subtropical Fruit Biology and Genetic Resource Utilization (MOA), Guangdong Province Key Laboratory of Tropical and Subtropical Fruit Tree Research, Guangzhou 510640, China; xiangdandan@gdaas.cn (D.X.); wenwen901203@hotmail.com (S.L.); yiganjun@vip.163.com (G.Y.); 3 MOE Key Lab for Intelligent Networks & Network Security, Faculty of Electronic and Information Engineering, Xi’an Jiaotong University, Xi’an 710049, China; wh513352261@stu.xjtu.edu.cn; 4 The School of Life Science and Technology, Xi’an Jiaotong University, Xi’an 710049, China

**Keywords:** *Fusarium oxysporum* f.sp. *cubense*, Fusarium wilt of banana, mycotoxin, prediction model

## Abstract

Fusarium wilt caused by *Fusarium oxysporum* f.sp. *cubense* (*Foc*) is one of the most destructive diseases for banana. For their risk assessment and hazard characterization, it is vital to quickly determine the virulence of *Foc* isolates. However, this usually takes weeks or months using banana plant assays, which demands a better approach to speed up the process with reliable results. *Foc* produces various mycotoxins, such as fusaric acid (FSA), beauvericin (BEA), and enniatins (ENs) to facilitate their infection. In this study, we developed a linear regression model to predict *Foc* virulence using the production levels of the three mycotoxins. We collected data of 40 *Foc* isolates from 20 vegetative compatibility groups (VCGs), including their mycotoxin profiles (LC-MS) and their plant disease index (PDI) values on Pisang Awak plantlets in greenhouse. A linear regression model was trained from the collected data using FSA, BEA and ENs as predictor variables and PDI values as the response variable. Linearity test statistics showed this model meets all linearity assumptions. We used all data to predict PDI with high fitness of the model (coefficient of determination (R^2^ = 0.906) and adjust coefficient (R^2^_adj_ = 0.898)) indicating a strong predictive power of the model. In summary, we developed a linear regression model useful for the prediction of *Foc* virulence on banana plants from the quantification of mycotoxins in *Foc* strains, which will facilitate quick determination of virulence in newly isolated *Foc* emerging Fusarium wilt of banana epidemics threatening banana plantations worldwide.

## 1. Introduction

*Fusarium oxysporum*, a major pathogen of many important crops worldwide, causes vascular wilt diseases in over 150 plant species [1,2]. Among which, Fusarium wilt of banana, caused by *F. oxysporum* f. sp. *cubense* (*Foc*), now prevalent in all banana growing regions, is considered the most destructive disease of banana [3]. Thousands of hectares of plantations have been devastated worldwide and serious threats have been posed to multi-billion dollar industry and to the stability of millions of farmers [4,5,6]. For instance, it was reported that an annual loss of more than 138 million dollars to the banana industry was caused due to Fusarium wilt in Australia alone [7].

As a typical soil-borne pathogen that produces chlamydospores as a resting propagule, *Foc* can persist in soil for a long time before infecting once it perceives the cues from banana roots [8,9]. The control of Fusarium wilt of banana relies on integrated strategies including growing resistant banana varieties, bio-control agents and crops rotations [8,10,11]. No fungicide is currently available to efficiently control the disease once plants are infected [12]. A lack of effective resistant plant cultivars and the absence of fungicides with high activity make the disease control extremely challenging. Therefore, a quick and reliable determination of pathogen virulence and disease severity is particularly important to differentiate high virulence isolates from mild or avirulent ones, which is critical to inform growers in disease management. With an urgent need to know the disease circumstances in real time or in advance, much efforts were invested to develop disease prediction models [13]. However, until now, there is still no model that can predict the occurrence and/or virulence of Fusarium wilt of banana precisely resulting from sampling bias, quality of occurrence data [14].

Research into the mechanisms of Fusarium pathogenicity has led to the identification and characterization of several secondary metabolites such as mycotoxins that contribute to disease progression in various plant diseases [15]. Fusarium species produce an extraordinary diversity of biologically active mycotoxins during the infection process [15]. Mycotoxin is one of the best studied virulence factors of phytopathogenic fungi [16]. Fusaric acid (FSA), beauvericin (BEA) and enniatins (ENs) were identified in *Foc* to contribute to pathogenicity during infection of host plants [17,18,19,20,21]. FSA is a non-host-specific toxin produced by all *Fusarium* species and plays a direct role in pathogenesis activity by disturbing the metabolism of the infected plant, leading to the inhibition of defensive enzymes and the reduction of cell viability of the host plants [22,23,24,25]. For Fusarium wilt of banana, FSA plays a critical role in accelerating the development of this disease by acting as a phytotoxin through disturbing the leaf water balance and nitrogen metabolism in the host banana plants [18,21]. BEA and ENs are structurally-related cyclic hexadepsipeptides belonging to the enniatin antibiotic family [26,27]. They are biosynthesized by more than 20 *Fusarium* species with a wide variety of biological properties such as insecticidal, antimicrobial, antiviral activities in vitro [28,29] The phytotoxic activities of BEA and ENs have been identified in different kinds of crops by inducing ascorbate system imbalance, oxidative stress [30], and depolarized electric potential [31,32]. Therefore, each of these mycotoxins is positively associated with fungal virulence. The disease severity is usually positively related to mycotoxin levels. It has been widely accepted that mycotoxins were produced simultaneously with fungal growth, and the rate of production is proportional to its growth rate. Several studies have already reported the modelling approach with mycotoxins in winter to predict fungal growth in different crops. For example, mycotoxins such as deoxynivalenol, zearalenone or aflatoxins were used to predict the Fusarium head blight in winter wheat [33] and *Aspergillus flavus* and *Aspergillus parasiticus* in black peppercorns [34]. Taken together, the characterization and quantification of the three mycotoxins could be useful for the prediction of fungal virulence, a major contributor to disease severity.

In this study, we aim to develop a statistical model to reliably predict the virulence of *Foc* isolates based on the production of three myctoxins, FSA, BEA and ENs. We first characterized and quantified *in vitro* mycotoxin production of 40 different strains of *Foc*. Next, we developed a linear regression model using mycotoxins (FSA, BEA and ENs) production as predictor variables and disease index as response variable. Lastly, we validated the predictive performance of the model by applying the trained model on new datasets.

## 2. Results and Discussion

### 2.1. Mycotoxin Quantified in Foc Isolates of Different VCGs

The production of three mycotoxins, BEA, FSA and ENs is substantial and could be detected in the *Foc* strains of 20 VCGs investigated in this study (Table 1). FSA production of the 40 isolates ranged from 1.30 ± 0.27 to 189.35 ± 9.76 μg/g. BEA production of the 40 isolates ranged from 1.38 ± 0.34 to 90.78 ± 9.22 μg/g. ENs production of the 40 isolates ranged from 0.58 ± 0.14 to 188.45 ± 28.70 μg/g. The mycotoxins produced by *Foc* play vital roles in the pathogenic process and show high frequencies of occurrence in food and feed. FSA is produced by the genus *Fusarium* and was reported to be detected in 85% of the swine feed samples with the highest concentration of 136 mg/kg [35]. It was also reported to increase the sensitivity of banana leaves and pseudostems to *Foc* TR4 invasion and acts at the early stage of the disease development before the appearance of the fungal hyphae in the infected tissues [36]. For BEA, the maximum reported concentrations for BEA in grains and in cereal-based food were 6400 and 844 μg/kg, respectively [37]. Also, ENs production variability *in vitro* was observed within a *F. avenaceum* population isolated from different hosts [38]. Our results are, in general, in agreement with other recently published studies, confirming that even if the structure of the two types of mycotoxins was similar, ENs contamination levels were considerably higher than BEA ones [39]. Likewise, a survey conducted on Italian cereal products and multicereal food showed that the maximum contamination levels detected were 1100 mg/kg for ENs and 70 mg/kg for BEA [40,41]. In conclusion, the range in these three mycotoxins production that we observed in our 40 isolates samples has shown to vary widely. These results coincide with those reported in literature which also showed that the ability of the different strains from different *Fusarium* species to produce mycotoxins varied widely [42]. It is reported that the viability of banana protoplasts was reduced to less than 20% after treatment with BEA at concentrations of 50 and 200 mM for 48 h and banana pseudostems treated with FSA and BEA became eroded *in vitro* in a concentration dependent manner [19]. Besides, simultaneous occurrence of BEA, FSA and ENs tested in our study is in agreement with other studies reporting co-contaminations of different mycotoxins [42,43,44]. For example, metabolites FSA, BEA, fumonisin (FB1) and moniliformin (MON) were isolated and quantified from rice plants infected with *Fusarium proliferatum* and among the four fungal metabolites, the productions of MON and FSA were found to have positive relationship with bakanae disease symptoms development [44]. Therefore, the pathogenicity of *Foc* may not be attributed to a single mycotoxin, but likely was a result of a synergy of several mycotoxins. Mycotoxins play significant roles in virulence, development, and overall lifestyle of the fungal pathogen. A closer investigation of the mycotoxin biology will give some practical benefits like understanding the nature of plant resistance to fungal diseases and will also be essential for the sustainable control of this important plant pathogen group [45].

### 2.2. Developing a Linear Regression Model for Prediction Foc virulence on Banana

**Figure 1:**
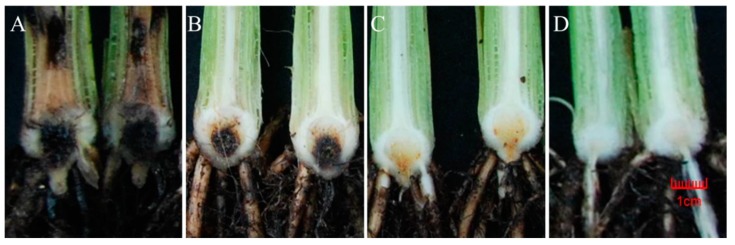
Classification of different pathogenicity of *Foc* isolates. (**A**) highly virulent: MAL10; (**B**) moderately virulent: GM; (**C**) weak virulent: GD-42; (**D**). Control.

**Figure 2:**
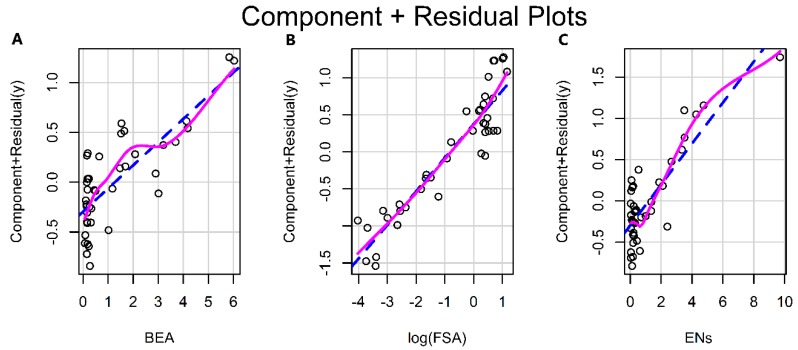
The component plus residual plots of each independent variable indicates three variables. (**A**) BEA, (**B**) log transformed FSA, (**C**) ENs.

All three mycotoxins have phytotoxic activity and all tested isolates proved to be producers and this made us to suspect their involvement in the expression of symptoms on banana plant. Therefore, the plant disease index (PDI) values of the same isolates were determined in Pisang Awak banana plantlets along with the mycotoxin productions being quantified in the isolates. As shown in Table 1, all the tested *Foc* isolates were able to infect banana plants and cause typical symptoms of Fusarium wilt of banana (Figure 1). Data showed that the disease severities and mycotoxin levels strongly differed between the *Foc* isolates. Xu suggested that there is a significant relationship between mycotoxins and the amount of FHB pathogen *F. graminearum* [46]. Likewise, Isolates MAL6, MAL10 and FJ-12 were highly virulent and caused the highest disease virulence to Pisang Awak with the PDI values of 4.80, 4.53 and 3.50. Isolates GD-15, GX-04 and GD-06 exhibited the lowest disease virulence on Cavendish banana plants with the PDI value of 0.43, 0.47 and 1.07, respectively. A good correlation between the PDI value and mycotoxin contents was found, that is, when a plant is severely infected, it is also heavily contaminated with mycotoxins. Thus, the *in vitro* assay is a useful tool to predict the possible mycotoxin contamination under field and greenhouse conditions [47]. What about the other way around? Could mycotoxin production levels of a *Foc* isolate be used to predict the virulence of the pathogen? A linear model for the prediction of disease virulence was developed in R programming language [48] based on mycotoxin and disease index (PDI) measurement of *Foc* isolates (Table 1). In this model, PDI value reflective of fungal virulence under controlled condition in lab was used as response variable, and the three mycotoxins FSA, BEA and ENs were used as predictor variables (Figure 2). The graphical residual analysis was performed, and it was found that the distribution between PDI and FSA, instead of BEA and ENs, are closer to logarithmic rather than linear distribution (Supplementary Figure S1). Hence, in order to improve calibration curve fit, a logarithmic conversion of the FSA variable was employed in this model (Figure 2B). The log transformation would decrease the distance between the lowest and highest point in the calibration range, thus leading to a more compressed regression line [49].

In building the model, we also consider the importance of mycotoxin toxicity in addition to its concentration, since it is not uncommon that a highly toxic compound is not necessarily produced in a high amount *in vitro* or *in planta*. Therefore, in order to generate a robust calibration model, the banana cell suspension EC_50_ (60 µM for FSA, 15 µM for BEA and 20 µM for ENs) was taken into account in the model by adjusting each variable with the EC_50_: log (C_FSA_/EC50_FSA_), C_BEA_/EC50_BEA_ and C_ENs_/EC50_ENs_. The resulting equation of the linear model is as follows:

(1) $$\mathrm{PDI}=0.23260\times \frac{C_{\mathrm{BEA}}}{\mathrm{EC}50_{\mathrm{BEA}}}+0.45392\times \log\left(\frac{C_{\mathrm{FSA}}}{\mathrm{EC}50_{\mathrm{FSA}}}\right)+0.24794\times \frac{C_{\mathrm{ENs}}}{\mathrm{EC}50_{\mathrm{ENs}}}+2.19782$$

The EC_50_ values indicated that all three kinds mycotoxins of were toxic to banana protoplast. This is in line with previous reports in which BEA caused white and dried up in the pseudostems, and black eroded upper tissue of banana plants, and at the same time, BEA is significantly more toxic than FSA [19]. It also confirmed that fungal metabolites possess vital role in disease development [18,19,21]. Moreover, mycotoxins are simultaneously produced by *Foc* in banana, and it is important that their additive and synergistic effects in the pathogenic process need to be further investigated in future.

### 2.3. Evaluating the Model Performance in PDI Prediction

Among the statistical models, linear regression models have shown promising results for its reasonable accuracy and relatively simple implementation [50]. A robust regression model is the foundation for accurate and reproducible quantification over the whole calibration range [49]. The coefficient of determination (R^2^) is one of the most important evaluation criteria of the quality for fiting the linear model to a given set of observed data. All the three variables had high coefficient (R^2^ = 0.9029) and adjusted coefficient (R^2^_adj_ = 0.8948), suggesting that the model explains more than 89.48% of the variability of the observations. A *p*-value of 2.2 × 10^−16^ for F-statistic implies that the linear relationship is statistically significant, and the model fits the data significantly better than the mean. Besides, a global test of the linear model assumption was performed by using the gvlma tool (package in R) to verify all the statistical constrains required by the linear model, including assumptions on skewness and kurtosis of the residual distribution, link function (linear model statistically significant) and heteroscedasticity (the variance of the residuals is dependent on the independent variable) [51]. The results from the global test (gvlma) confirm that the linear model assumptions are all satisfied with *p* value higher than > 0.05 (Table 2).

**Figure 3:**
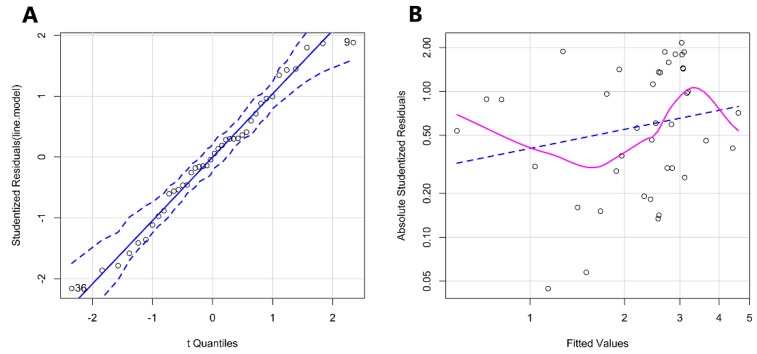
(**A**) The Q-Q plot of all data. (**B**) Absolute Studentized Residuals of dataset. All the data are in a range of −2–2 expect one point. And these two plots illustrate that this dataset follows normal distribution.

Quantile-quantile (Q-Q) plot is also used to evaluate the normal assumption of the residuals which compares the observed residual distribution to theoretical one by plotting their quantile against each other [52,53]. As suggested in Figure 3A, the error terms can generally be considered as normally distributed and the absolute studentized residual plot (Figure 3B) showed that the differences between predicted and observed data in initial datasets are in a range of −2–2, except one point, indicating that all data follow a normal distribution.

Finally, to evaluate the performance of this linear model in prediction, it was used to predict the PDI using an input of new dataset collected from Pathogenicity assay on Pisang Awak cv ‘Guangfen #1’ (ABB) banana plantlets, which include both mycotoxin contents in the diseased plantlets and the PDI values. As shown in Table 3, the differences between predicted and observed PDI are in a range of −0.3–0.6 which proves that our model performed well.

Certainly, this model also has uncertainties resulting from sampling bias and the data quality. Due to the limited size of the data points used to fit the model, the obtained results are contextual. Plant disease outcome is dependent on various factors such as pathogen virulence, host resistance, environmental factors including temperature, humidity and microbiomes. Given such a complex nature of plant disease development, it is extremely challenging to accurately predict the disease occurrence and severity in field conditions. Our work does not attempt to predict plant disease epidemics in the field, which requires a more sophisticated model of many variables, probably non-linear or nonparametric nature. Instead, we focus on developing a simple yet powerful model allowing a quick determination of fungal virulence in *Foc*, the causal agent of Fusarium wilt of banana, to facilitate the risk assessment and help implementing disease control strategies. By taking the advantage of positive correlation of mycotoxin production to disease index, we trained a linear regression model using training data from 40 *Foc* isolates including mycotoxin and disease index measurement. Applying the model to testing data demonstrates the predictive power of such model. Admittedly, the model is simple taking into account only three variables, and probably could be improved by incorporating more variables such as fungal effectors, more mycotoxins etc. However, this would require additional inputs such as quantification of more toxins or genotyping of the strains in more loci, a process that would risk overfitting the model or slow down the report of prediction results. Therefore, the current model gives a reliable prediction of *Foc* virulence in a short time within a couple of weeks. With a better knowledge of the fungal virulence through experimental analysis of genes or genetic pathways, it is conceivable that the model can be further improved to use factors so that it either requires even less data collection time or gives higher accuracy of prediction.

## 3. Conclusions

In this study, the levels of mycotoxins were used to develop a model to predict fungal virulence, which can be used as a tool for assessing the risk of newly isolated *Foc* strains from environment. Fusarium wilt of banana epidemics are usually tightly linked to the *Foc* inoculum and its ability to produce *Foc* mycotoxins. The results reported here extended our knowledge of suitable indicators of *Foc* infection and mycotoxin production. The current model is quite simple and primitive, only the production ability of three mycotoxins known to contribute to virulence in *Foc* is taken into account. Nevertheless, this study provides preliminary information that might be useful for future risk analysis. With more mycotoxins or effectors contributing to fungal virulence identified in the future, the model can be improved by incorporating more variables representing these virulence factors. However, we believe a predictive model is sorely needed now to help differentiate highly virulent *Foc* strains from only mild or avirulent ones, so that risks of newly emerged *Foc* strains can be assessed, and quick and timely actions can be taken for disease control.

## 4. Materials and Methods

### 4.1. Chemicals and Reagents

Analytical standards FSA, BEA and ENs with purities ≥97% were purchased from Sigma-Aldrich (St. Louis, MO, USA). Acetonitrile (ACN), methanol (MeOH), methanoic acid and other organic solvent (chromatographic-grade) were obtained from Fisher Scientific (Fair Lawn, NJ, USA). Stock solutions of these mycotoxins (1 mg/mL) were prepared in methanol and maintained at −20 °C. Final concentrations of mycotoxins in the assay were achieved by their dilution in the culture medium and the final concentration of methanol in the medium was 0.5% (v/v). Water was purified successively by a Millipore Milli-Q system (Millipore, Bedford, MA, USA) with a conductivity < 18.2 MΩ.cm at 25 °C.

### 4.2. Fungal Strain and Plant Material

*Foc* monoconidial isolates (VCGs 0120–01220) used for the mycotoxin production in this study are all maintained in PDA (potato dextrose agar medium, Guangdong Huankai Microbial SCI. & Tech. Co. LTD., Guangzhou, China) with 15% glycerol at −80 °C at the Agricultural Culture Collection of China (ACCC) and confirmed by pathogenicity tests on banana Baxi (*Musa* AAA Cavendish) and Guangfen No.1 (*Musa* ABB Pisang Awak) (Table 1). The fungal strains were routinely inoculated onto the center of 90 mm plates and were incubated at 28 °C for 7 days. These fresh cultures were used to prepare inoculum for mycotoxin production analysis. Banana cultivars (Baxi (*Musa* AAA Cavendish) and Guangfen No.1 (*Musa* ABB PisangAwak)) with 5–6 leaves used in the experiments were provided by the Banana Tissue Culture Center, Institute of Fruit Tree Research, Guangdong Academy of Agriculture Science.

### 4.3. Extraction of Mycotoxin Produced by Foc In Vitro

*Foc* isolates were inoculated onto PDA plates and cultured in the dark at 28 °C. After 15 days, the mycelia were harvested, sonicated and the metabolites were extracted with methanol. The extracts were analyzed by high performance liquid chromatography-tandem mass spectrometry (HPLC-ESI-MS; LCQDECA XP PLUS; Thermo Finnigan, Thermo Corp., Rockford, IL, USA). Detailed protocols for chemical extraction, instrumental conditions, and quality assurance/quality control (QA/QC) were provided in the Supplementary Materials. Mycotoxins used as standards included FSA, BEA, ENs.

### 4.4. Toxicity of Mycotoxin to Banana

Banana protoplasts were employed in this study to determine the toxic effects of mycotoxins produced by *Foc* to banana. The banana protoplasts used in this study were obtained from embryogenic cell suspensions (ECS) of the cultivar ‘Dongguan Dajiao’ (ABB) as described previously (Table S1) [19,54]. Brief protocols for the ECS initiation and maintenance are provided in Supplementary Materials. The viable protoplasts were isolated from the ECS at a yield of 1.2 × 10^7^ protoplasts/mL packed cell volume (PCV) and suspended in 20 mL of pre-plasmolysis buffer (25 mM Tris–Mes, 0.6 M sorbitol, 0.5% BSA and 0.5% CaCl_2_, pH 7.4) and exposed to different concentrations of FSA, BEA and ENs (0, 2, 10, 20 50, 100 and 200 μM, n = 3). The toxicity effects of the three mycotoxins to banana protoplasts were conducted after 3 days’ culture using the Alamar Blue assay according to manufacturers’ recommendations (Biotium, Hayward, CA, USA). Briefly, the reactions were performed in 96-well plate (Costar, Cambridge, MA, USA) with 2 × 10^4^ cells contained (in 200 μL culture medium) in each well and 20 μL Alamar Blue (10% v/v) was added and the cells were further incubated for another 4 h in an incubator at 37 °C. Then the fluorescence was measured (excitation 530 nm, emission 590 nm) with a Spectra Max i3 plate reader (Molecular Devices, Sunnyvale, CA, USA). At least triplicate wells were analyzed for each experiment.

### 4.5. Pathogenicity Assays

In order to study the relationship between mycotoxin biosynthesis and pathogenicity, 8 VCGs (Table 1) were randomly selected to conduct the virulence assay on Pisang Awak cv ‘Guangfen #1’ (ABB) banana plantlets at the 5- to 6-leaf stage in the sterilized planting medium (six parts vermiculite, two parts peat, and one part coconut coir) for pathogenicity studies. The plants were inoculated with spore suspensions of the 8 VCGs at a concentration of 10^5^ conidia/g sterilized planting medium and all the inoculated plants were kept in a greenhouse with the temperature ranging from 25–28 °C and the humidity was set at 40%, with the soil moisture maintained at about 60%. Observations on the virulence of wilt disease on banana plantlets were scored after 30 days inoculation with a disease index based on a 1–5 scale [55]. The percent disease index (PDI) was calculated as follows: Disease index = ∑(rating × number of plants rated/Total number of plants). And at the same time, a group with no *Foc* treatment served as control. Treatments were replicated three times in completely randomized block design with each replication consisted of 10 plantlets (20 g). Plant tissues were collected and subjected to homogenization with the blender (Ultra-Turrax T18, IKA, Germany) for 3 min and subjected to mycotoxin extraction per the methods described in Section 4.3.

### 4.6. Development and Validation of Prediction Model for Fusarium Wilt of Banana

A predictive linear model regarding the risk of *Foc* pathogenicity was developed using R packages [48], gvlma [51] and car [56]. The contents of three mycotoxins (FSA, BEA and ENs) measured in the 40 *Foc* strains and the PDI values obtained from pathogenicity assays were selected for the model development (Table 1). A linearity test was used between each mycotoxin and PDI value to guarantee the accuracy of the model. Since EC_50_ is an important index to assess the virulence, the ratio between each mycotoxin concentration and its EC_50_ was used as predictor variables in the linear model and at the same time the PDI value as response variable. The graphical residual analysis was performed for each mycotoxin and logarithmic transformations were applied on FSA variable because the distribution between FSA and PDI are closer to logarithmic rather than linear distribution. The model contains the following general equation for the prediction of PDI of Fusarium wilt of banana is established as follow:

y = a + b × log(C_FSA_/EC50_FSA_) + d × C_BEA_/EC50_BEA_ + e × C_ENs_/EC50_ENs_ (2)

where a, b, d, and e are the parameters to be estimated, and C_FSA_, C_BEA_ and C_ENs_ stand for the concentrations of FSA, BEA, and ENs. In addition, EC50_FSA_, EC50_BEA_, and EC50_ENs_ stand for the phytotoxicity of FSA, BEA, and ENs respectively.

### 4.7. Statistical Analysis and Data Processing

All statistical analyses of the data were performed in the statistical program package SPSS 20.0 (SPSS, Chicago, IL, USA). Data were initially verified for normality and homogeneity of variance by using the Kolmogorov-Smirnov and Levene’s tests, respectively. All data were reported as means ± standard error of the mean (SEM). Differences between the control and each exposure group were evaluated by one-way analysis of variance (ANOVA) followed by Tukey’s test. If ANOVA revealed significant effects of treatments, the data were subjected to Tukey’s post-hoc test for statistical significance (*p* < 0.05).

## Figures and Tables

**Table 1:** *Fusarium oxysporum* f. sp. *Cubense* isolates used in this study and their mycotoxin production *in vitro* and their ability to cause diseases in Pisang banana plantlets.

Strains ^a^	VCGs	Race	Provider	Fusaric Acid Concentration (μg/g) ^b,c^	Beauvericin Concentration (μg/g) ^b,c^	Enniatins Concentration (μg/g) ^b,c^	Disease Index
GD-14	0120	STR4	Chunyu Li	164.98 ± 2.23^B^	24.51 ± 2.53^F^	2.93 ± 0.73^L^	3.50
GD-42	0120	STR4	Chunyu Li	17.66 ± 3.66^MNO^	15.56 ± 3.56^HJ^	3.41 ± 1.17^KL^	1.50
GM	0121	TR4	Chunyu Li	189.35 ± 9.76^A^	2.58 ± 1.41^JK^	4.47 ± 1.30^JKL^	3.00
F9130-2	0121	TR4	Randy C. Ploetz	1.94 ± 0.54^P^	2.43 ± 1.11^JK^	188.45 ±28.70^A^	2.73
CAV443	01210	STR4	Altus Viljoen	78.20 ± 0.46^HIJ^	45.18 ± 4.34^D^	2.37 ± 0.41^L^	2.53
CAV632	01210	STR4	Altus Viljoen	135.44 ± 11.03^C^	2.35 ± 0.57^JK^	14.93 ± 4.80^IJ^	2.30
SH3142	01211	STR4	Randy C. Ploetz	23.43 ± 1.28^MN^	17.27 ± 1.89^GH^	41.54 ± 4.21^FG^	2.53
SH3142-3	01211	STR4	Randy C. Ploetz	11.70 ± 1.85^OP^	24.27 ± 1.96^F^	37.11 ± 2.28^G^	2.37
STTNZ1	01212	1	Randy C. Ploetz	1.30 ± 0.27^P^	2.26 ± 0.76^JK^	72.44 ± 14.16^D^	1.80
STNP2-3	01212	1	Randy C. Ploetz	1.42 ± 0.30^P^	3.53 ± 0.66^JK^	96.45 ± 6.43^B^	2.03
GD-06	1213	TR4	Chunyu Li	2.85 ± 1.63^P^	2.41 ± 1.44^JK^	0.58 ± 0.14^L^	1.07
FJ-11	1213	TR4	Chunyu Li	4.73 ± 1.85^P^	3.82 ± 1.25^JK^	3.90 ± 1.24^KL^	1.13
MW2	01214	1	Randy C. Ploetz	118.77 ± 5.78^D^	1.84 ± 0.26^K^	2.38 ± 0.37^L^	2.60
MW40	01214	1	Randy C. Ploetz	86.54 ± 2.15G^H^	2.40 ± 1.44^JK^	4.97 ± 0.75^JKL^	2.30
01215-M	01215	STR4	Chunyu Li	103.44 ± 4.47^E^	1.38 ± 0.34^K^	1.14 ± 0.46^L^	2.13
1215-1	01215	STR4	Chunyu Li	118.66 ± 7.20^D^	3.40 ± 0.16^JK^	0.91 ± 0.28^L^	2.17
GD-13	01216	TR4	Chunyu Li	75.13 ± 10.79^IJ^	22.11 ± 1.23^FG^	8.36 ± 3.65^JKL^	2.83
FJ-12	01216	TR4	Chunyu Li	95.40 ± 5.40^EFG^	66.21 ± 17.06^B^	20.33 ± 3.02^HI^	3.50
MAL10	01217	1	Randy C. Ploetz	84.82 ± 2.82^GH^	90.78 ± 9.22^A^	53.67 ± 2.58^E^	4.53
MAL6	01217	1	Randy C. Ploetz	89.47 ± 3.13^FGH^	87.70 ± 3.54^A^	71.44 ± 6.64^D^	4.80
GX-04	01218	1	Randy C. Ploetz	2.14 ± 1.15^P^	1.55 ± 0.65^K^	3.67 ± 6.64^KL^	0.47
STSUM3	01218	1	Randy C. Ploetz	9.58 ± 0.80^OP^	2.37 ± 0.22^JK^	0.95 ± 0.40^L^	1.37
INDO25-1	01219	STR4	Randy C. Ploetz	13.42 ± 2.07^NOP^	4.97 ± 0.80^JK^	6.19 ± 0.78^JKL^	1.63
INDO25-2	01219	STR4	Randy C. Ploetz	27.66 ± 0.25^M^	6.85 ± 0.50^JK^	1.22 ± 0.56^L^	2.07
PW6	0122	STR4	Randy C. Ploetz	72.80 ± 7.14^J^	31.15 ± 3.14^E^	6.12 ± 1.16^JKL^	2.93
PW7	0122	STR4	Randy C. Ploetz	88.98 ± 4.34^FGH^	43.49 ± 2.22^D^	7.37 ± 1.17^JKL^	2.87
GD-15	01220	1	Chunyu Li	1.68 ± 0.50^P^	2.47 ± 1.19^JK^	3.58 ± 0.37^KL^	0.43
HN-03	01220	1	Chunyu Li	2.87 ± 0.47^P^	7.52 ± 1.75^JK^	6.97 ± 0.82^JKL^	1.13
HN-11	0123	1	Chunyu Li	119.99 ± 8.50^D^	31.88 ± 9.10^E^	1.35 ± 0.22^L^	3.43
GX-02	0123	1	Chunyu Li	173.00 ± 26.15^B^	22.86 ± 1.84^FG^	4.14 ± 1.15^JKL^	3.50
GD-37	0124	1	Chunyu Li	5.63 ± 0.41^P^	61.44 ± 0.78^B^	26.70 ± 1.29^H^	2.37
HN-13	0124	1	Chunyu Li	11.66 ± 1.64^OP^	2.32 ± 0.62^JK^	2.53 ± 0.48^L^	1.53
CAV941	0125	1	Altus Viljoen	119.55 ± 2.14^D^	2.84 ± 0.62^JK^	10.50 ± 1.95^IJKL^	3.23
CAV125	0125	1	Altus Viljoen	100.28 ± 5.39^EF^	9.59 ± 0.31^IJ^	1.22 ± 0.19^L^	3.00
GD-18	0126	STR4	Chunyu Li	4.29 ± 0.59^P^	54.43 ± 1.46^C^	27.48 ± 2.52^H^	2.03
GD-26	0126	STR4	Chunyu Li	92.61 ± 3.75^EFG^	4.20 ± 0.84^JK^	47.87 ± 2.50^EF^	2.43
GX-03	0128	1	Cavendish	90.06 ± 13.47^FG^	4.69 ± 0.24^JK^	4.59 ± 1.26^JKL^	2.33
CAV 567	0128	1	Altus Viljoen	4.56 ± 0.48^P^	1.67 ± 0.43^K^	67.18 ± 2.89^D^	1.97
CAV186	0129	STR4	Altus Viljoen	46.50 ± 1.01^L^	2.67 ± 1.33^JK^	85.79 ± 3.87^C^	3.47
CAV 438	0129	STR4	Altus Viljoen	58.17 ± 1.67^K^	47.67 ± 4.68^D^	14.34 ± 1.27^IJK^	3.03
Control 1 ^d^				0	0	0	0
Control 2 ^e^				0	0	0	0

^a^ The *Foc* isolates were maintained at Agricultural Culture Collection of China (ACCC); ^b^ Data are expressed as mean ± standard error (mean ± SEM); ^c^ Mean values in the same column followed by the different capital letter are significantly different by Fisher’s protected least significant difference test (*p* < 0.05); ^d^ Control 1 is sterilized PDA medium; ^e^ Control 2 is sterilized deionized water.

**Table 2:** Summary of linear model hypothesis test.

Test	Value	*p*-Value	Decision
Global Stat	1.3483097	0.8531	Assumptions met
Skewness	0.0004423	0.9832	Assumptions met
Kurtosis	0.6439081	0.4223	Assumptions met
Link Function	0.5084728	0.4758	Assumptions met
Heteroscedasticity	0.1954865	0.6584	Assumptions met

**Table 3:** Additional dataset used as input for predicting PDI with the linear model.

Strains	FSA Concentration (μg/g) ^a^	BEA Concentration (μg/g) ^a^	ENs Concentration (μg/g) ^a^	Disease Index	Predicted Disease Index
GD30	4.65 ± 0.38	3.83 ± 0.19	2.22 ± 0.30	0.57	1.13
GD19	56.12 ± 4.33	4.46 ± 1.22	1.46 ± 0.45	2.03	2.26
GD20	5.49 ± 0.92	1.75 ± 0.55	2.16 ± 0.38	0.73	1.05
GD05	3.55 ± 0.53	2.62 ± 0.12	2.27 ± 0.26	0.60	0.94
GX01	100.57 ± 1.80	17.68 ± 1.98	2.59 ± 0.56	3.00	2.72
HN04	11.72 ± 1.71	6.48 ± 0.56	1.63 ± 0.45	1.57	1.62
FJ10	13.39 ± 1.29	4.64 ± 0.46	4.49 ± 0.55	1.20	1.60
YN6	9.14 ± 0.67	4.72 ± 0.58	2.37 ± 0.27	1.37	1.43
Control ^b^	0	0	0	0	0

^a^ Data are expressed as mean ± standard error (mean ± SEM); ^b^ The control in this experiment was the healthy Pisang Awak cv ‘Guangfen #1’ (ABB) banana plantlets treated with sterilized deionized water.

## Supplementary Materials

The following are available online at https://www.mdpi.com/2072-6651/12/4/254/s1, Table S1: The ingredient list for M1 and M2 medium, Figure S1: The component plus residual plots of each independent variable. The more fitted the purple and blue dotted lines, the more linear. And if a plot is non-linear, it means insufficient functional modeling of predictors and may do some transform.
