# Supplementary material for: Predicting Virulence of Fusarium oxysporum f. sp. Cubense Based on the Production of Mycotoxin Using a Linear Regression Model
Source: Toxins (Basel). 2020 Apr 14;12(4):254. doi: 10.3390/toxins12040254 (PMC7232494; doi:10.3390/toxins12040254)
Supplement: Supplementary file 1 [file toxins-12-00254-s001.pdf]

Supplementary Materials: Predicting Virulence of *Fusarium Oxysporum* f. sp. *Cubense* Based on the Production of Mycotoxin Using a Linear Regression Model

Chuange Shao, Dandan Xiang, Hong Wei, Siwen Liu, Li Guo, Chunyu Li, Shuxia Lyu and Ganjun Yi

Mycotoxin Identification and Quantification

The identification and quantification of FSA, BEA, and ENs were performed with a LC-MS/MS system using methods described published previously [1]. In brief, The chromatographic separation of the compounds was conducted at 24 ± 1 °C on a reverse-phase analytical column Gemini® C18 (3 μm, 150 × 2 mm i.d.) and a guard-column C18 (3 μm, 4 × 2 mm, i.d.;) from Phenomenex (Madrid, Spain). The mobile phases were employed as follows: 95% water, 5% acetonitrile and 0.1% formic acid (v:v:v) as Phase A and 95% acetonitrile, 5% water and 0.1% formic acid (v:v:v) as Phase B. And the following gradient was used: equilibration at 90% A for 2 min, from 80% to 20% A in 3 min, 20% A for 1 min, from 20% to 10% A in 2 min, 10% A for 6 min, from 10% to 0% A in 3 min, 100% B for 1 min, from 100% to 50% B in 3 min, return to initial conditions in 2 min, and maintain at initial conditions for 2 min. The injection volume was 20 μL with the flow rate of 0.20 mL/min in all steps.

Initiation and Maintenance of Banana Embryogenic Cell Suspensions (ECS)

Briefly, the immature male flowers were excised and cultured in the M1 (Table S1) medium for callus induction for 5–6 months and then the friable embryogenic calli were selected and subsequently cultured in liquid M2 medium (Table S1) to initiate cell suspensions [2,3]. Homogeneous and yellow ECS were obtained after sieving selection of the cultures using a stainless mesh with pore sizes of 154 mm at 15 day-intervals for 3 months. The stable ECS used in this study were cultured on a rotary shaker (100 rpm) at 27 °C in the dark and passaged every 2 weeks with a 1.5% (v/v) dilution in 100 ml Erlenmeyer flasks containing 30 ml M2 medium.

Table S1. The ingredient list for M1 and M2 medium.

Medium	MS	4.1 μM biotin	18 μM 2,4-D	5.7 μM IAA
	**5.4 μM NAA**	**87 mM sucrose**	**7 g/Lagarose**	
M2 medium	MS	4.1 μM biotin	4.5 μM 2,4-D	680 μM glutamine
	100 mg/Lmalt extract	130 mM sucrose		
MS medium	1.90 g/L Potassium nitrate	1.65 g/L Ammonium nitrate	440 mg/L Calcium chloride·2H_2O	180.69 mg/L Magnesium sulphate
	170 mg/L Potassium phosphate monobasic	16.90 mg/L Manganese sulphate.H_2O	6.20 mg/L Boric acid	0.83 mg/L Potassium iodide
	0.25 mg/L Molybdic acid (sodium salt)·2H_2O	8.60 mg/L Zinc sulphate·7H_2O	0.025 mg/L Copper sulphate·5H_2O	0.025 mg/L Cobalt chloride·6H_2O
	27.80 mg/L Ferrous sulphate·7H_2O	37.30 mg/L EDTA disodium salt·2H_2O	100 mg/L Myo-Inositol	0.10 mg/L Thiamine hydrochloride
	0.50 mg/L Pyridoxine hydrochloride	0.50 mg/L Nicotinic acid (Free acid)	2.00 mg/L Glycine (Free base)	
M1 medium	MS	4.1 μM biotin	18 μM 2,4-D	5.7 μM IAA
	5.4 μM NAA	87 mM sucrose	7 g/L agarose	
M2 medium	MS	4.1 μM biotin	4.5 μM 2,4-D	680 μM glutamine
	100 mg/L malt extract	130 mM sucrose		

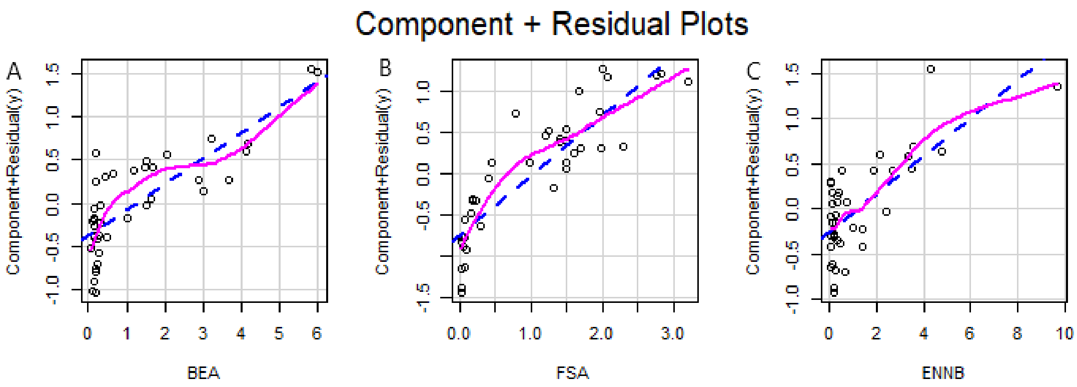

Figure S1. The component plus residual plots of each independent variable. (**A**) BEA, (**B**) FSA, (**C**) ENs. The more fitted the purple and blue dotted lines, the more linear. And if a plot is non-linear, it means insufficient functional modeling of predictors and may do some transform.

References

1. Stanciu, O.; Juan, C.; Miere, D.; Loghin, F.; Mañes, J. Analysis of enniatins and beauvericin by LC-MS/MS in wheat-based products. *Cyta-J Food* **2017**.*15*, 433-440.
2. Escalant, J.V.; Teisson, C.; Cote, F. Amplified somatic embryogenesis from male flowers of triploid banana and plantain cultivars (*Musa spp*.). *In Vitro Cell Dev-Pl* **1994**. *30*, 181-186.
3. Côte, F.X.; Domergue, R.; Monmarson, S.; Schwendiman, J.; Teisson, C.; Escalant, J.V. Embryogenic cell suspensions from the male flower of Musa AAA cv. Grand nain. *Physiol Plantarum* **1996**. *97*, 285-290.
